# Investigating chromosomal instability in long-term survivors with glioblastoma and grade 4 astrocytoma

**DOI:** 10.3389/fonc.2023.1218297

**Published:** 2024-01-08

**Authors:** Jochem K. H. Spoor, May den Braber, Clemens M. F. Dirven, Adam Pennycuick, Jirina Bartkova, Jiri Bartek, Vera van Dis, Thierry P. P. van den Bosch, Sieger Leenstra, Subramanian Venkatesan

**Affiliations:** ^1^ Department of Neurosurgery, Brain Tumor Center, Erasmus University Medical Center, Rotterdam, Netherlands; ^2^ Department of Paediatric Neurosurgery, Erasmus Medical Center Sophia Children’s Hospital, Rotterdam, Netherlands; ^3^ Lungs for Living Research Centre, UCL Respiratory, Division of Medicine, University College London, London, United Kingdom; ^4^ Genome Integrity Group, Danish Cancer Institute, Danish Cancer Society, Copenhagen, Denmark; ^5^ Division of Genome Biology, Department of Medical Biochemistry and Biophysics, Science for Life Laboratory, Karolinska Institute, Stockholm, Sweden; ^6^ Department of Pathology, Erasmus University Medical Center, Rotterdam, Netherlands; ^7^ Department of Oncology, University College London, London, United Kingdom

**Keywords:** high grade glioma, glioblastoma multiforme, astrocytoma, chromosomal instability, genome instability, IDH

## Abstract

**Background:**

Only a small group of patients with glioblastoma multiforme (GBM) survives more than 36 months, so-called long-term survivors. Recent studies have shown that chromosomal instability (CIN) plays a prognostic and predictive role among different cancer types. Here, we compared histological (chromosome missegregation) and bioinformatic metrics (CIN signatures) of CIN in tumors of GBM typical survivors (≤36 months overall survival), GBM long-term survivors and isocitrate dehydrogenase (IDH)-mutant grade 4 astrocytomas.

**Methods:**

Tumor sections of all gliomas were examined for anaphases and chromosome missegregation. Further CIN signature activity analysis in the The Cancer Genome Atlas (TCGA)-GBM cohort was performed.

**Results:**

Our data show that chromosome missegregation is pervasive in high grade gliomas and is not different between the 3 groups. We find only limited evidence of altered CIN levels in tumors of GBM long-term survivors relative to the other groups, since a significant depletion in CIN signature 11 relative to GBM typical survivors was the only alteration detected. In contrast, within IDH-mutant grade 4 astrocytomas we detected a significant enrichment of CIN signature 5 and 10 activities and a depletion of CIN signature 1 activity relative to tumors of GBM typical survivors.

**Conclusions:**

Our data suggest that CIN is pervasive in high grade gliomas, however this is unlikely to be a major contributor to the phenomenon of long-term survivorship in GBM. Nevertheless, further evaluation of specific types of CIN (signatures) could have prognostic value in patients suffering from grade 4 gliomas.

## 1 Introduction

Glioblastoma multiforme (GBM), defined by the fifth edition of the World Health Organization (WHO) classification of tumors of the central nervous system as an isocitrate dehydrogenase 1/2 (*IDH1/2*) wild type (WT) diffuse glioma, is the most common and aggressive tumor of the central nervous system (1). GBM requires multimodal treatment strategies consisting of surgery, chemotherapy, and radiotherapy. Despite this, patients typically have a dismal prognosis with a median survival of around 15 months, from now on referred to as GBM typical survivors (GBM-TS). There is, however, a minority of GBM patients that have a survival of more than 36 months, so-called GBM long-term survivors (GBM-LTS) (2). Several clinical factors have been associated with long-term survivorship, including a good initial performance status, absence of focal neurological deficits, extensive surgical resection, young age and female sex (2–5). Additionally, tumor genomic factors have been studied in this context and these studies have linked long-term survivorship with higher levels of aneuploidy (6), MGMT promoter methylation (2, 7), *IDH1/2* mutation (7), p53 overexpression (8) and loss of 19q (9). Additionally, less proliferation showed a trend toward significance in one study (4) and was significantly associated with long-term survivorship in a larger study (8). To improve the overall survival of GBM patients, it is vital to better understand the underlying basis of long-term survival in these patients.

Genome instability is considered an enabling characteristic that underlies the acquisition of other cancer hallmarks (10). Chromosomal instability (CIN) is a specific form of genome instability and refers to the elevated rate of gaining and losing whole-chromosomes or segments of chromosomes (11), which occurs through chromosome missegregation. This missegregation happens during mitosis, specifically during anaphase, and can be observed as chromatin bridges and lagging chromosomes. Chromatin bridges predominantly arise from pre-mitotic defects such as replication stress and defective DNA repair, whereas lagging chromosomes are thought to result from errors that arise during mitosis such as defects in sister chromatid cohesion and kinetochore-microtubule attachments (12, 13). Although there are other mechanisms of chromosome missegregation, in general a high frequency of lagging chromosomes and chromatin bridges is a strong indicator of CIN (14). Direct observation of anaphases in hematoxylin and eosin (H&E)-stained tissue sections using light microscopy, provides insights into the extent of CIN *in vivo*. This method has previously been used to associate CIN with tumor prognosis in another cancer type (15).

Different studies report both adverse and beneficial effects of CIN on patient prognosis or response to therapy. Depending on factors such as tumor origin and the therapeutic context, CIN can be either tumor suppressive or oncogenic. For example, in patients with diffuse large B-cell lymphoma, an increased rate of CIN was associated with poor prognosis (15). In contrast, in patients with locally invasive rectal adenocarcinoma, CIN was associated with a favorable response to chemoradiation therapy (16). Studies have shown that higher rates of CIN promote genetic heterogeneity in tumor cell populations, thereby providing a substrate for selection and tumor evolution (17), enabling adaptation to treatments and accelerated relapse.

In this study, we investigate chromosomal instability in tumors of GBM-TS, GBM-LTS and IDH-mutant (MUT) grade 4 astrocytomas using histological and bioinformatic methods.

## 2 Methods

### 2.1 Study population

Two patients with too few evaluable anaphases, defined as less than 15, were excluded from further analysis, leaving 20 patients within our study. The final study population includes 10 GBM-TS, 5 GBM-LTS and 5 patients with IDH-MUT grade 4 astrocytomas. Patients were retrospectively selected from a patient cohort treated at the neurosurgery department at Erasmus University Medical Center. Patients were included in this study if they met the following criteria: 1) they were diagnosed with primary glioblastoma in the period of 2005 to 2015 as adults, 2) tissue from the first tumor resection was available, 3) patients were not treated pre-operatively for high grade glioma. Additionally, long-term survivor patients had to have an overall survival of at least 36 months. Patient data such as sex, age, treatment, and survival were obtained through a hospital database. Overall survival was calculated by the period from the first tumor resection to February 2022, or death. Tumors containing an IDH mutation were reclassified as an IDH-MUT grade 4 astrocytoma in accordance with the latest edition of the WHO classification of tumors of the central nervous system.

### 2.2 Assessing chromosomal missegregation

**Figure 1:**
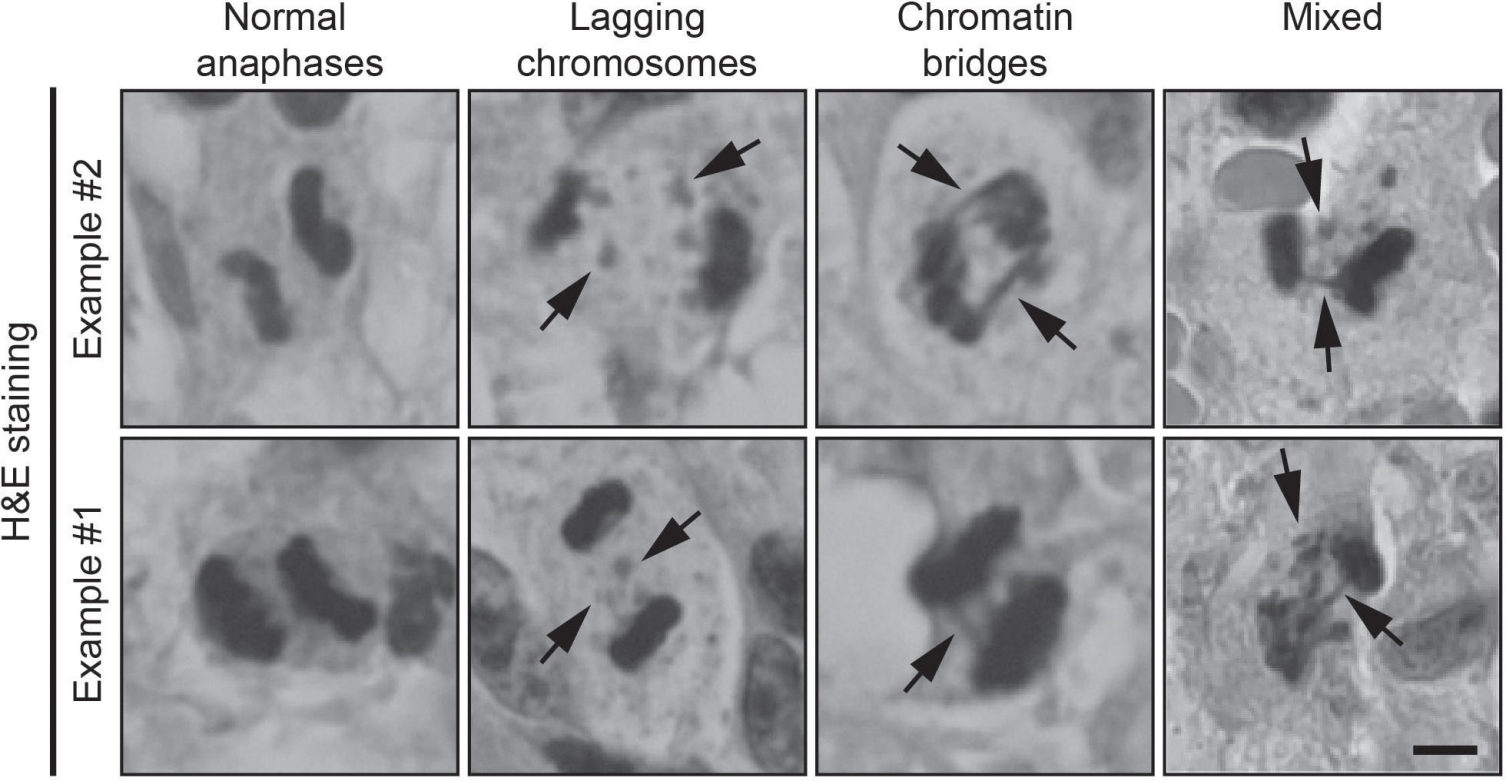
Examples of H&E-stained samples of normal anaphases, lagging chromosomes (arrows) and chromatin bridges (arrows). Scale bar, 5 μm.

The tumor tissue was initially assessed by a neuropathologist to ensure that examinations were done on representative tumor material. For the examination of chromosome missegregation, 4 μm thick slices were made from formalin-fixed paraffin-embedded samples and standard H&E staining was performed using the Ventana HE600 (Roche Diagnostics). Sections were examined using a 40x and 100x oil immersion objective on an Olympus BX50 light microscope. Using the 40x objective, the tissue sections were analyzed for cells undergoing anaphase. The number of cells in anaphase was calculated per mm^2^ of tumor tissue. All cells in anaphase were scored for evidence of chromosome missegregation using the 100x oil immersion objective. The cells in anaphase were classified as one of the following: normal (N), lagging chromosome (LC), chromatin bridge (CB) or mixed (M) (**Figure 1**). Anaphase cells were not evaluated or included in the analyses if the sister chromatids were too close to each other to be able to observe lagging chromosomes or chromatin bridges. Non-evaluable cells were not included in the final analyses. Lagging chromosomes were defined as an area of hematoxylin staining stranded in between segregating sister chromatids. Chromatin bridges were defined as at least one continuous band of hematoxylin staining linking the remaining segregating sister chromatids. Cells in anaphase were considered ‘normal’ in the absence of both lagging chromosomes and chromatin bridges. When both lagging chromosomes and chromatin bridges were found in 1 anaphase cell, it was labeled as ‘mixed’. For the final analysis of our data, the classification of the assessed cells was simplified to either ‘normal’ or undergoing ‘chromosome missegregation’. Images were created on a Zeiss Axiophot microscope with an Olympus DP25 camera using a 40x objective.

### 2.3 Immunohistochemistry

#### 2.3.1 Determining *IDH1* mutation status

The *IDH1* mutation status was retrieved via a hospital database or determined using IDH1 R132H antibody staining. Following deparaffinization and heat-induced antigen retrieval, the tissue samples were incubated with anti-IDH1 (1/800, clone H09, Dianova) visualized with Ultraview Red (#760-501, Ventana) and stained with hematoxylin counter stain and a blue coloring reagent. Tumor tissue samples that were *IDH1* mutant stained red, while *IDH1*-WT tumors remained blue. Since the IDH1 R132H antibody staining has high sensitivity and specificity (18) together with *IDH1* R132H mutations comprising ~91% of all *IDH1* mutations (19), we are confident we have detected almost all *IDH1* mutant tumors in our cohort.

#### 2.3.2 p16 and p53 Immunohistochemistry

Sequential 4 µm thick (FFPE) sections were stained for p16 and p53 using Ultraview universal DAB detection Kit (#760-700, Ventana). In brief, following deparaffinization and heat-induced antigen retrieval with CC1 (#950-500, Ventana) for 64 minutes, tissue samples were incubated with either anti-p16 (1.0 µg/ml ready to use, clone E6H4, Ventana) or anti-p53 (2.5 µg/ml ready to use, clone BP53-11, Ventana) for 32 minutes at 37°C. Incubation was followed by a hematoxylin II counter stain for 8 minutes and then a blue coloring reagent for 8 minutes according to the manufacturer’s instructions (Ventana).

#### 2.3.3 (phosphorylated) pRPA-S33, pRPA-S4/S8 and γH2AX immunohistochemistry

To detect the replication stress-related phosphoprotein markers in human glioblastoma specimens, we employed our well-established sensitive immunohistochemical staining protocol (20). Standard deparaffinization of the archival formalin-fixed, paraffin-embedded tissue sections was followed by antigen unmasking in Tris/EDTA buffer (pH 9, 15 minutes microwave exposure). After overnight incubation with primary antibodies (mouse monoclonal antibody to human histone, H2A.X phosphorylated on Ser 139, Millipore,Temecula, CA, USA, clone JBW 301,diluted 1:2500; rabbit polyclonal antibody to human RPA2 phosphorylated on Ser33, Novus Biologicals, batch NB 100-544, diluted 1:20 000; and rabbit polyclonal antibody to human RPA2 phosphorylated on Ser4/Ser8, Novus Biologicals, batch NB P1 23017, diluted 1:1500) the sections were processed for the indirect streptavidin-biotin-peroxidase method using the Vectastain Elite kit (Vector Laboratories) and nickel-sulphate-based chromogen enhancement detection, as described previously, without nuclear counterstaining (20, 21). For negative controls, human glioblastoma sections were incubated with non-immune serum instead of the primary antibodies, followed by identical subsequent detection steps. The results were evaluated by two experienced researchers, including a senior oncopathologist, and the data was expressed based on the percentage of positive tumor cells expressing the respective phosphoprotein marker (see examples of staining patterns in **Supplementary Figure 3B**).

### 2.4 Data analysis

A log-rank (Mantel-Cox) test was performed to assess differences in overall survival. A one-way ANOVA with a *post-hoc* Dunnett’s multiple comparisons test was performed to evaluate statistical differences between tumors of GBM-TS, GBM-LTS and IDH-MUT grade 4 astrocytomas, if the data was normally distributed with a homogeneity of variances across groups. A Kruskal-Wallis test with a *post-hoc* Dunn’s test was performed to evaluate differences between the 3 different groups, if data normality or homogeneity of variances was not present. A two-tailed unpaired *t* test was performed to compare 2 groups with normally distributed data. A Mann-Whitney test was performed to compare 2 groups with non-normally distributed data. A Fisher’s exact test was performed when comparing categorical variables. A p-value (*P*) and False Discovery Rate (*FDR*) ≤ 0.05 were considered significant (α=0.05), whereas p-values and FDR-values > 0.5 were considered non-significant.

### 2.5 Bioinformatics analysis

Level 3 pre-processed data were obtained from The Cancer Genome Atlas (TCGA) Data Portal using the TCGAbiolinks R/Bioconductor package (22). The functions GDCquery, GDCdownload and GDCprepare were used to import data from the “TCGA-GBM” cohort into R (http://www.r-project.org) for further analysis. Data were downloaded in June 2023.

Methods regarding the CIN signature can be found in (23). In short, only samples were considered for which copy number segment data, mutation data and metadata (i.e., “days_to_death” and “days_to_last_follow_up”) were specified. Samples were excluded if the copy number segment data did not pass quality control. We used the CINSignatureQuantification bioinformatics package (23). The scaled activities of the 17 CIN signatures were derived after which data were rescaled (minimum=0, maximum=1). A Kruskal-Wallis test with a *post-hoc* Dunn’s test was performed and p-values were adjusted for multiple testing by using the Benjamini-Hochberg method.

### 2.6 Weighted genome integrity index

The weighted genome integrity index (wGII) was calculated for each TCGA sample following established methods (24, 25). Level 3 copy number segment data was downloaded from Genomic Data Commons. The wGII was then calculated for each sample as the proportion of the genome with copy number not equal to estimated ploidy, corrected such that each chromosome is equally weighted.

### 2.7 Data availability statement

Data generated by the Cancer Genome Atlas (TCGA) pilot project were downloaded. The TCGA was established by the NCI and the National Human Genome Research Institute. Information about The TCGA and the investigators and institutions who constitute the TCGA research network can be found at https://cancergenome.nih.gov/. The analysis code has been deposited in GitHub at https://github.com/manivenkatesan/SpoorEtAl_CINsignatures.

## 3 Results

To assess the presence of CIN in tumors of GBM-TS, GBM-LTS and IDH-MUT grade 4 astrocytomas, we observed anaphase cells in H&E-stained tissue sections using light microscopy. Sections from 20 patients were examined for evidence of chromosome missegregation. A median of 35 evaluable anaphases (range, 18-108) was found among all samples.

### 3.1 Patient and tumor characteristics

**Figure 2:**
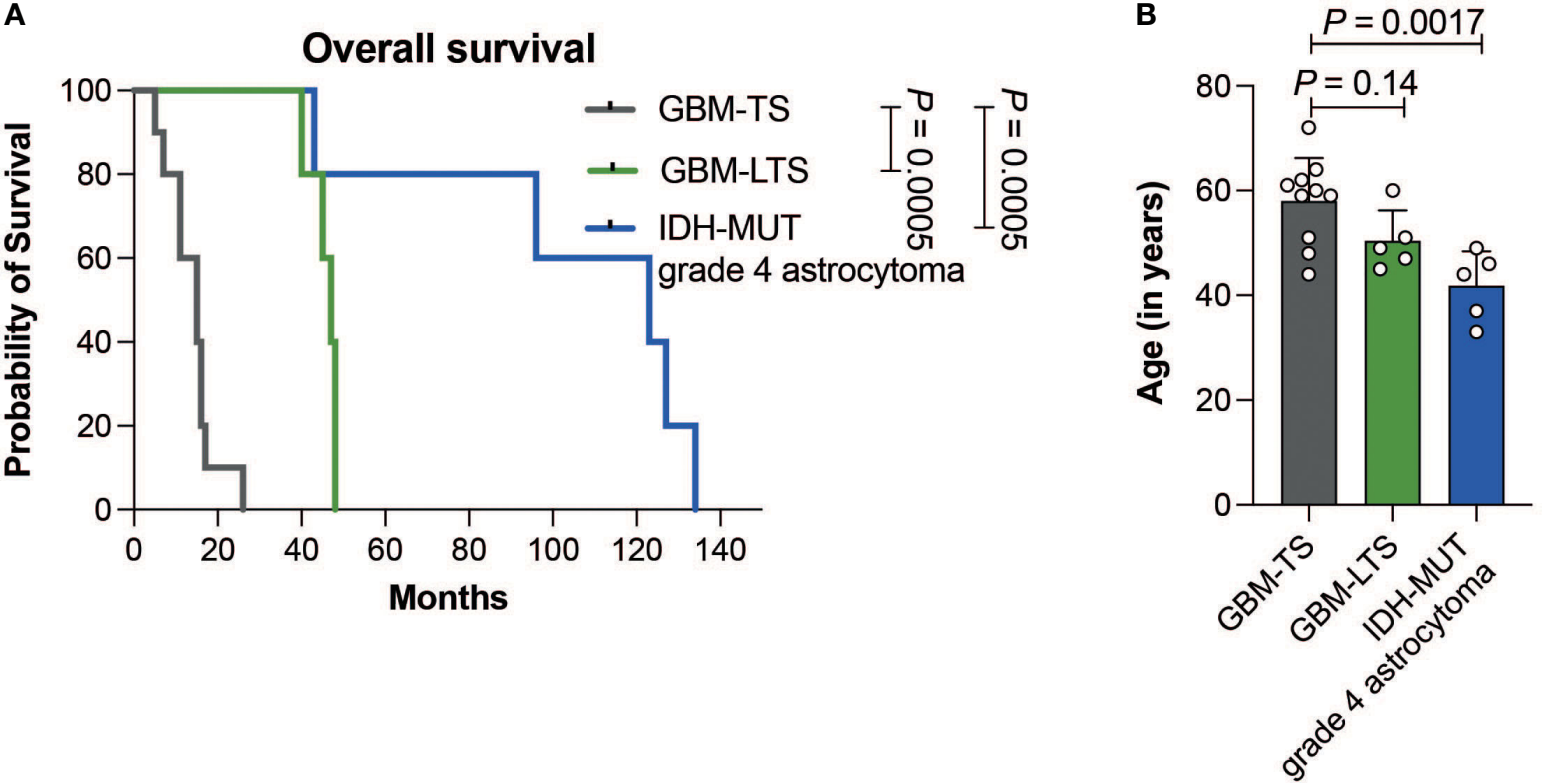
**(A)** Kaplan-Meier curve showing the difference in overall survival between GBM-TS (n = 10, grey), GBM-LTS (n =5, green) and IDH-MUT grade 4 astrocytoma patients (n = 5, blue) (log-rank test, *P =* 0.0005). **(B)** Barplots comparing the patients’ age in years. Results represent mean ± SD (one-way ANOVA with Dunnett’s multiple comparisons test).

GBM-TS patients (n = 10) had a median survival of 15 ± 5.9 months (median ± SD, range, 5-26) and median age of 60 years (range, 44-72 years). GBM-LTS patients (n = 5) had a median survival of 47 ± 3.4 months (median ± SD; range, 40-48) with a median age of 49 years (45-60 years). IDH-MUT grade 4 astrocytoma patients (n = 5) had a median survival of 123 ± 37.3 months (median ± SD; range, 43-134) with median age of 44 years (33-49 years). A list of patient and tumor characteristics is shown in **Supplementary Table 1**. Patients with IDH-MUT grade 4 astrocytomas had significantly longer overall survival relative to GBM-TS and GBM-LTS patients (log-rank test, *P =* 0.0005, **Figure 2A**). Additionally, patients with IDH-MUT grade 4 astrocytomas were significantly younger relative to GBM-TS (one-way ANOVA with Dunnett’s multiple comparisons test, *P* = 0.0017, **Figure 2B**). In an attempt to further characterize our cohort, we performed p16 and p53 immunohistochemistry (**Supplementary Figure 1A**). We observed significantly fewer samples with loss of p16 expression in IDH-MUT grade 4 astrocytomas relative to tumors of GBM-TS (Fisher’s exact test, *P* = 0.044, **Supplementary Figure 1B**), whereas there were no differences between the 3 groups in p53 overexpression (Fisher’s exact test, *P* > 0.05, **Supplementary Figure 1C**).

### 3.2 Histological measure of CIN in grade 4 glioma patients

**Figure 3:**
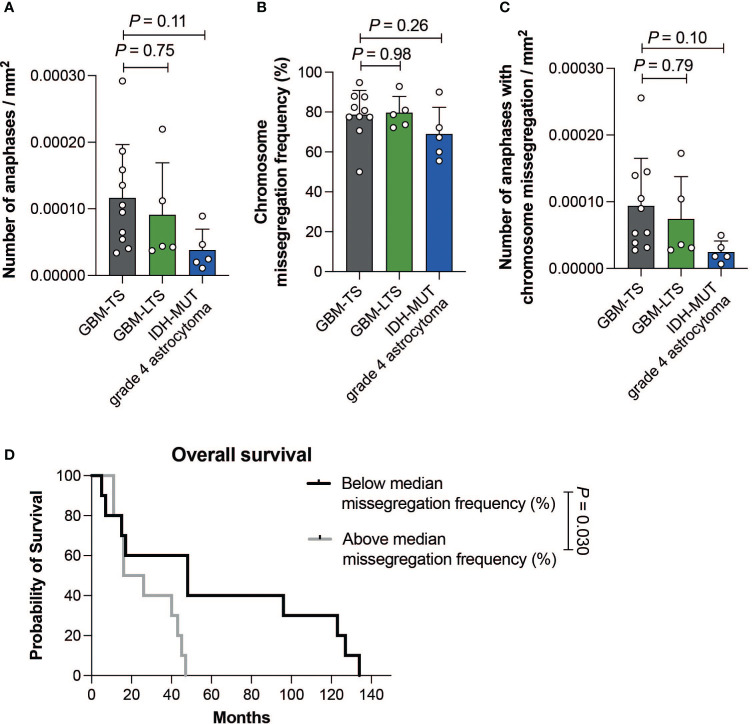
**(A)** Number of anaphases per mm^2^ of tumor tissue. Results represent mean ± SD (one-way ANOVA with Dunnett’s multiple comparisons test). **(B)** Chromosome missegregation frequency of cells in anaphase. Results represent mean ± SD (one-way ANOVA with Dunnett’s multiple comparisons test). **(C)** Number of cells in anaphase exhibiting chromosome missegregation per mm^2^ of tumor tissue. Results represent mean ± SD (one-way ANOVA with Dunnett’s multiple comparisons test). **(D)** Kaplan-Meier curve showing the difference in overall survival between patients with a chromosome missegregation frequency below (n = 10, black) and above the median (n =10, grey) (log-rank test, *P =* 0.030).

Within GBM-TS, GBM-LTS and IDH-MUT grade 4 astrocytoma tumor sections, we respectively observed a median of 50 anaphases (range, 24-108), 28 anaphases (range, 18-75) and 20 anaphases (range, 18-55). There were no significant differences in the number of cells in anaphase per mm^2^ of tumor tissue (one-way ANOVA with Dunnett’s multiple comparisons test, *P* > 0.05, **Figure 3A**). All groups showed an overall high chromosome missegregation frequency, with a range of 50.0% - 94.8% (**Figure 3B**). The median chromosome missegregation frequency in GBM-TS, GBM-LTS and IDH-MUT grade 4 astrocytoma tumor sections were respectively 79.2% (range, 50.0%-94.8%), 78.7% (range, 72.2%-92.9%) and 67.3% (range, 55.6%-90.0%). There were no significant differences in chromosome missegregation frequencies between the 3 groups (one-way ANOVA with Dunnett’s multiple comparisons test, *P* > 0.05, **Figure 3B**) and also not if the cohort was stratified according to p16 or p53 immunohistochemical staining patterns (respectively, Mann-Whitney test and unpaired *t* test, *P* > 0.05, **Supplementary Figures 2A, B**).

Furthermore, there were no significant differences in the number of cells in anaphase with chromosome missegregations per mm^2^ between the 3 groups (one-way ANOVA with Dunnett’s multiple comparisons test, *P* > 0.05, **Figure 3C**). Next, we investigated whether CIN could be prognostic within grade 4 glioma patients regardless of their survivor status. We divided our patient cohort according to the median chromosome missegregation frequency (median = 77.6%). Interestingly, the group with a chromosome missegregation frequency below the median had significantly longer survival than the group above the median (log-rank test, *P =* 0.030, **Figure 3D**). In summary, we did not find differences in chromosome missegregation between tumors of GBM-TS, GBM-LTS and IDH-MUT grade 4 astrocytomas, however we did find evidence for chromosome missegregation frequencies providing prognostic information for grade 4 glioma patients.

### 3.3 Differences in CIN signatures relative to gliomas of GBM-TS

**Figure 4:**
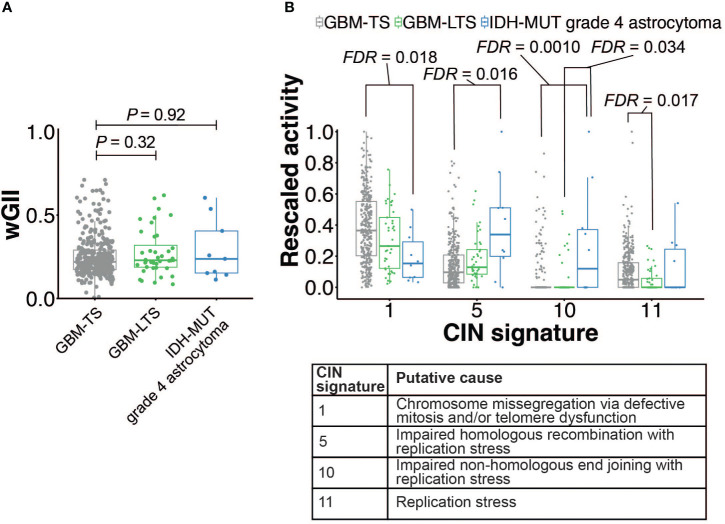
**(A)** Comparison of wGII scores between GBM-TS (n = 346, grey), GBM-LTS (n =40, green) and IDH-MUT grade 4 astrocytoma patients (n = 9, blue) (Kruskal-Wallis test with Dunn’s *post hoc* test, corrected for multiple testing by using the Benjamini-Hochberg method, *P* > 0.05). **(B)** Relative activities of the 4 significantly altered CIN signatures (GBM-TS, n = 354; GBM-LTS, n = 41; IDH-MUT grade 4 astrocytoma, n = 10). Boxplots summarize rescaled CIN signature activities (Kruskal-Wallis test with Dunn’s *post hoc* test, corrected for multiple testing by using the Benjamini-Hochberg method, *FDR* < 0.05). Boxes represent the interquartile range with the median depicted as a bold line and the whiskers extend to 1.5 times the interquartile range from the lower or upper quartile, with datapoints outside this interval being considered as potential outliers.

To assess CIN in tumors of GBM-TS, GBM-LTS and IDH-MUT grade 4 astrocytomas within a larger dataset using an orthogonal method, we decided to interrogate the TCGA-GBM cohort. Initially, we compared the weighted genome integrity index (wGII) between the three groups, however we did not detect any significant differences (Kruskal-Wallis test with Dunn’s *post hoc* test, *P* > 0.05, **Figure 4A**). Rather than a global CIN metric, we next investigated whether specific types of CIN signatures (23). Drews and colleagues recently described 17 CIN signatures based on single-nucleotide polymorphism (SNP) array data from the TCGA and their “CINSignatureQuantification” package deconstructs the contribution of each CIN signature per sample (23). This cohort contained 354 GBM-TS, 41 GBM-LTS and 10 IDH-MUT grade 4 astrocytomas with appropriate copy number variation data. We employed the CINSignatureQuantification package (23) to quantify the activities of all 17 CIN signatures and looked for differences between the 3 groups. Interestingly, the only altered CIN signature in tumors of GBM-LTS relative to that of GBM-TS was a depletion of CIN signature 11 (Kruskal-Wallis test with Dunn’s *post hoc* test, *FDR* = 0.017, **Figure 4B**). Replication stress was previously assigned as a putative cause of CIN signature 11 (23). IDH-MUT grade 4 astrocytomas had 3 different CIN signature alterations relative to tumors of GBM-TS. CIN signature 1 activity was significantly lower in IDH-MUT grade 4 astrocytomas relative to tumors of GBM-TS (Kruskal-Wallis test with Dunn’s post hoc test, *FDR *= 0.018, **Figure 4B**). In contrast, CIN signature 5 and 10 activities were significantly higher in IDH-MUT grade 4 astrocytomas relative to tumors of GBM-TS (Kruskal-Wallis test with Dunn’s post hoc test, respectively *FDR* = 0.016 and *FDR* = 0.0010, **Figure 4B**). Chromosome missegregation via defective mitosis and/or telomere dysfunction were previously implicated in contributing to CIN signature 1, impaired homologous recombination with replication stress in CIN signature 5 and impaired non-homologous end joining with replication stress in CIN signature 10 (23). We did not detect significant changes in any of the other CIN signatures (Kruskal-Wallis test with Dunn’s *post hoc* test, respectively *FDR* > 0.05, **Supplementary Figure 3A**). To further investigate any differences in replication stress and double-strand breaks in our cohort, we performed immunohistochemistry of (phosphorylated) pRPA-S33, pRPA-S4/S8 and γH2AX (**Supplementary Figure 3B**). We did not detect any differences in the percentage of cancer cells positive for these markers (one-way ANOVA with Dunnett’s multiple comparisons test, **Supplementary Figures 3C-E**). In short, 1 CIN signature was altered in GBM-LTS relative to GBM-TS, whereas 3 CIN signatures were significantly altered in IDH-MUT grade 4 astrocytomas relative to GBM-TS.

## 4 Discussion

High grade glioma is the most aggressive type of primary brain cancer with a dismal prognosis. The median survival of GBM patients is around 15 months. Nevertheless, a small percentage survives beyond 36 months, so-called GBM long-term survivors (26, 27). This raises the question of what determines long-term survivorship. Here, we investigated histological and copy number variation-based measures of CIN among tumors of GBM-TS, GBM-LTS and IDH-MUT grade 4 astrocytomas. Recently, higher CIN levels have been linked to worse progression-free and overall survival in astrocytomas (28, 29). However, to our best knowledge, CIN has not been specifically investigated in relation to long-term survivorship (>36 months) within grade 4 glioma patients.

We compared chromosome missegregation rates by examining the presence of chromosomal missegregation in tumor tissue sections. As chromosome missegregation occurs during the anaphase of mitosis, we could directly observe these events in H&E-stained tissue sections using light microscopy. Interestingly, all gliomas in our cohort had a missegregation frequency of at least 50%, making the overall chromosome missegregation frequency generally higher compared to that of recent studies in other cancer types (15, 16, 21, 30). Since long-term survivors are uncommon in patients with GBM, we were only able to analyze tissue from a small set of patients. Classically, the percentage of anaphases with chromosome missegregations is used to obtain a measure of CIN within tumor sections (15, 30). Using this method, we do not see any differences in the percentage of anaphases with chromosome missegregations between tumors of GBM-TS, GBM-LTS and IDH-MUT grade 4 astrocytomas. Nevertheless, a split of our cohort by the median chromosome missegregation frequency revealed that the group with a chromosome missegregation frequency below the median had a significantly longer survival than the group above the median. Altogether, these data show that even though the phenomenon of GBM-LTS cannot specifically be explained by differences in chromosome missegregation, a histological measure of CIN can provide prognostic information for patients with grade 4 gliomas.

Recently, Drews and colleagues reported 17 signatures that characterize specific types of CIN (23). Our bioinformatic analysis revealed that tumors of GBM-LTS had significantly depleted CIN signature 11 activity relative to that of GBM-TS. Drews and colleagues previously attributed replication stress and slippage at poly T repeats during DNA replication as contributors to CIN signature 11 (23). We were unable to detect any difference in the extent of immunohistochemical replication stress markers within our cohort. A likely explanation for this result is that there are multiple genetic and epigenetic causes that collectively lead to replication stress in a given tumor, whereas immunohistochemistry only provides a snapshot of phosphoprotein expression that reflects the stress signaling at a given timepoint. Furthermore, it is still unclear whether CIN signature activities change in the course of cancer evolution. IDH-MUT grade 4 astrocytomas have significantly lower CIN signature 1 activity and higher CIN signature 5 and 10 activities relative to tumors of GBM-TS. CIN signature 1 was classified as a mitotic signature and associates with whole-arm or whole-chromosome changes and correlates with downregulation of telomerase activity (23). Additionally, CIN signature 1 positively correlates with single base substitutions signature 1 (SBS1) (23), suggesting that a natural aging process might contribute to CIN signature 1. Indeed, in our cohort, patients with IDH-MUT grade 4 astrocytomas are significantly younger than GBM-TS. Within our cohort we do not find a difference in age between GBM-LTS and GBM-TS, in contrast others have reported that GBM-LTS associates with a younger age at diagnosis (2–5). Interestingly, Drews and colleagues also noted a significant negative association between mutant *IDH1* and CIN signature 1 in a pan-cancer analysis (23), strengthening our observation in grade 4 gliomas. Impaired homologous recombination has been implicated in CIN signature 5. Interestingly, recent reports show that IDH mutant tumors have suppressed homologous recombination and are consequently sensitive to poly-ADP ribose polymerase (PARP) inhibitors (31, 32). Currently the implication and relevance of CIN signature 10 enrichment in IDH-MUT grade 4 astrocytomas relative to tumors of GBM-TS is unclear. Altogether, our data show limited evidence of altered levels of CIN in tumors of GBM long-term survivors relative to that of GBM typical survivors. In contrast, IDH-MUT grade 4 astrocytomas appear to have multiple alterations of specific CIN signatures relative to tumors of GBM typical survivors.

Our study has several limitations. Firstly, our cohort of GBM-LTS is small. This is mainly due to the rare occurrence of long-term survivors in high grade glioma patients, but it is also complicated by the fact that we need relatively large non-necrotic H&E sections to detect sufficient amounts of anaphases. Secondly, we cannot fully rule out differences in chromosome missegregation frequency per cell division between the 3 groups. Our analysis is complicated by low sample sizes and the general scarcity of anaphase cells found in glioma sections. Further investigation of CIN in *in vitro* and *in vivo* models of IDH-WT and IDH-MUT high grade gliomas will be needed. These models will provide an appropriate platform to investigate how the CIN status may inform prognosis and therapy decisions.

In summary, our data suggest that CIN is pervasive in high grade gliomas and is generally higher than that reported in other cancer types. Apart from a depletion in CIN signature 11, we do not find further evidence for CIN driving the phenomenon of GBM-LTS. Our data suggest that mutant IDH may partially confer a less aggressive tumor phenotype through the change of specific types of CIN, although other factors are also likely to play a role, such as the modulation of extracellular matrix stiffness (33). Further evaluation of different types of CIN and CIN signatures could have prognostic value in patients suffering from grade 4 gliomas.

## Data availability statement

The datasets presented in this study can be found in online repositories. The names of the repository/repositories and accession number(s) can be found in the article/**Supplementary Material**.

## Ethics statement

The studies involving humans were approved by The Erasmus MC Medical Ethics Review Committee. The studies were conducted in accordance with the local legislation and institutional requirements. The human samples used in this study were acquired as part of our previous study, for which ethical approval was obtained. Written informed consent for participation was not required from the participants or the participants’ legal guardians/next of kin in accordance with the national legislation and institutional requirements.

## Author contributions

JS, SL and SV developed the concept and wrote the first draft of the manuscript. JS, MB, CD, AP, JirinB, JiriB, VD, TB, SV collected and analyzed the data. All authors critically revised the manuscript. All authors contributed to the article and approved the submitted version.

## Supplementary material

Supplementary Figure 1
**(A)**, Examples of negative and positive immunohistochemical stains for IDH1 R132H, p53 and p16. Scale bar, 50 μm. **(B)**, Number of patients with tumors exhibiting loss of p16 expression or retained p16 expression (Fisher’s exact test). **(C)**, Number of patients with tumors exhibiting p53 overexpression or no p53 overexpression (Fisher’s exact test).

Supplementary Figure 2
**(A)**, Chromosome missegregation frequency of cells in anaphase stratified by p16 expression or retained p16 expression. Results represent mean ± SD (Mann-Whitney test). **(B)**, Chromosome missegregation frequency of cells in anaphase stratified by p53 overexpression or no p53 overexpression. Results represent mean ± SD (two-tailed unpaired *t* test).

Supplementary Figure 3
**(A)**, Relative activities of 13 CIN signatures that are not significantly different between the 3 groups (GBM-TS, n = 354; GBM-LTS, n = 41; IDH-MUT grade 4 astrocytoma, n = 10). Boxplots summarize rescaled CIN signature activities (Kruskal-Wallis test with Dunn’s *post hoc* test, corrected for multiple testing by using the Benjamini-Hochberg method, *FDR* > 0.05). Boxes represent the interquartile range with the median depicted as a bold line and the whiskers extend to 1.5 times the interquartile range from the lower or upper quartile, with datapoints outside this interval being considered as potential outliers. **(B)**, Examples of negative and positive immunohistochemical stains for pRPA-S33, pRPA-S4/S8 and γH2AX. Scale bar, 50 μm. **(C-E)**, Frequency of immunopositive cells. Results represent mean ± SD (one-way ANOVA with Dunnett’s multiple comparisons test).
