# Supplementary figures and images for: Investigating chromosomal instability in long-term survivors with glioblastoma and grade 4 astrocytoma
Source: Front Oncol. 2024 Jan 8;13:1218297. doi: 10.3389/fonc.2023.1218297 (PMC10800987; doi:10.3389/fonc.2023.1218297)

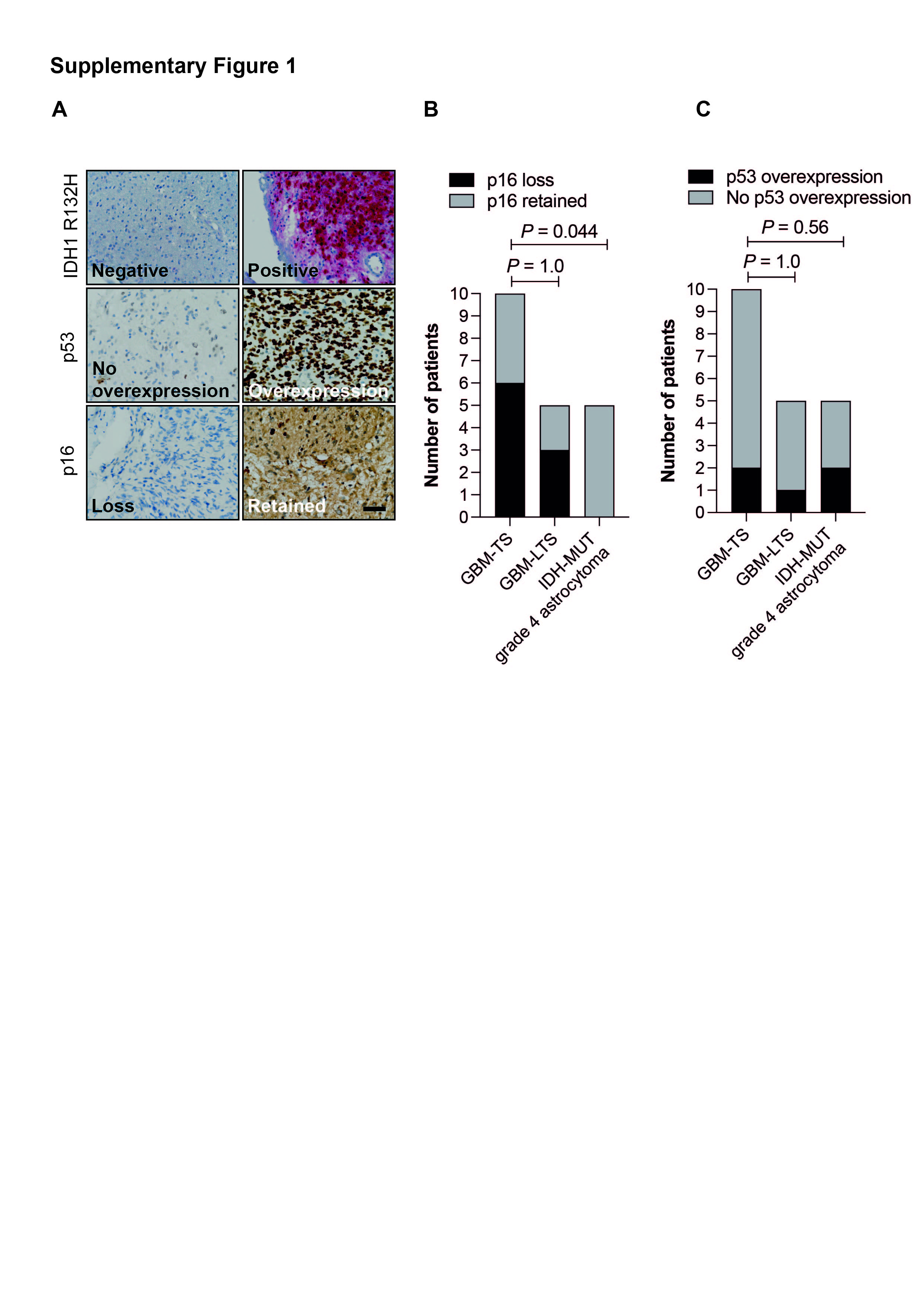

Supplement: Supplementary Figure 1 — (A), Examples of negative and positive immunohistochemical stains for IDH1 R132H, p53 and p16. Scale bar, 50 μm. (B), Number of patients with tumors exhibiting loss of p16 expression or retained p16 expression (Fisher’s exact test). (C), Number of patients with tumors exhibiting p53 overexpression or no p53 overexpression (Fisher’s exact test). [file Image_1.jpeg]

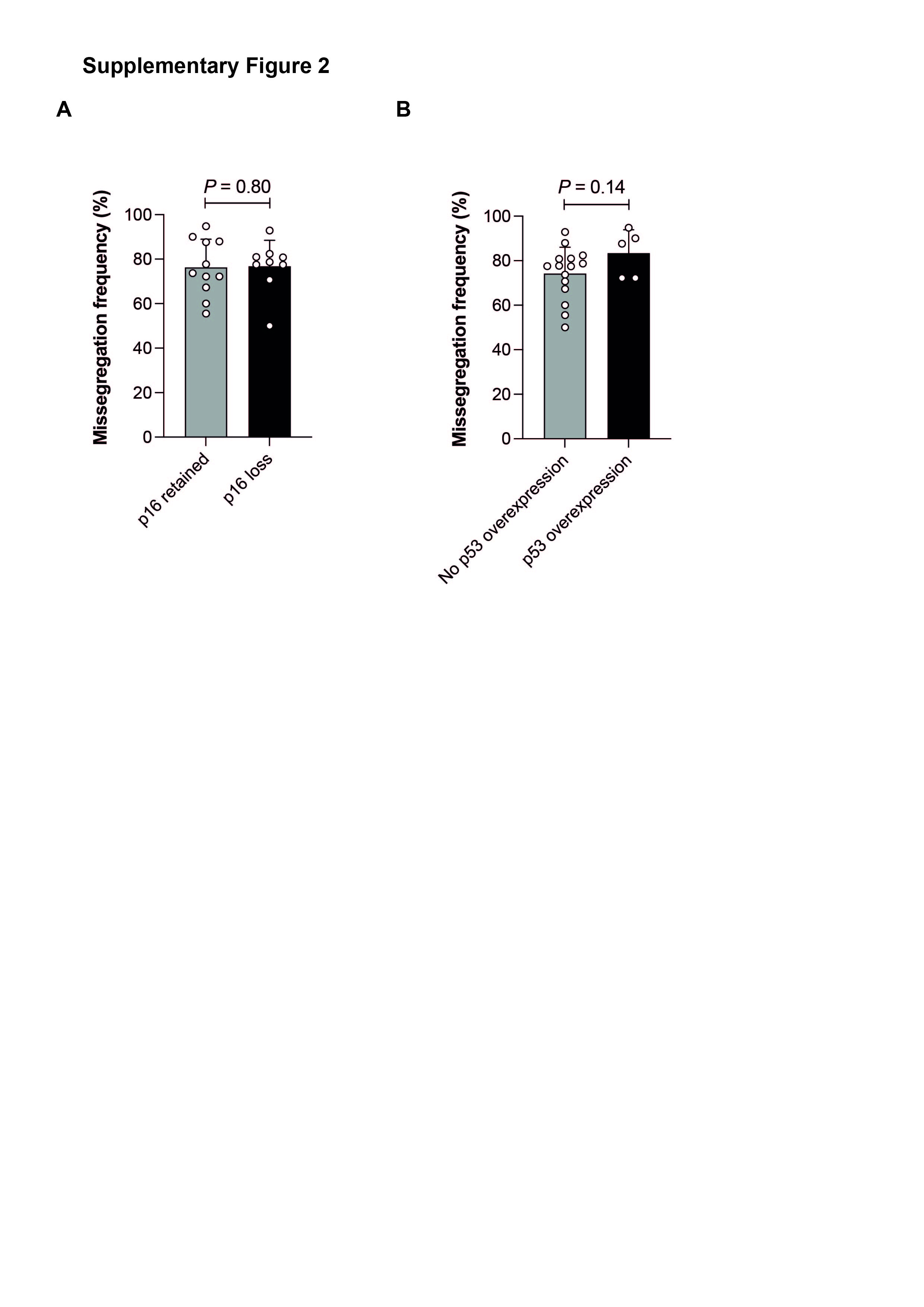

Supplement: Supplementary Figure 2 — (A), Chromosome missegregation frequency of cells in anaphase stratified by p16 expression or retained p16 expression. Results represent mean ± SD (Mann-Whitney test). (B), Chromosome missegregation frequency of cells in anaphase stratified by p53 overexpression or no p53 overexpression. Results represent mean ± SD (two-tailed unpaired t test). [file Image_2.jpeg]

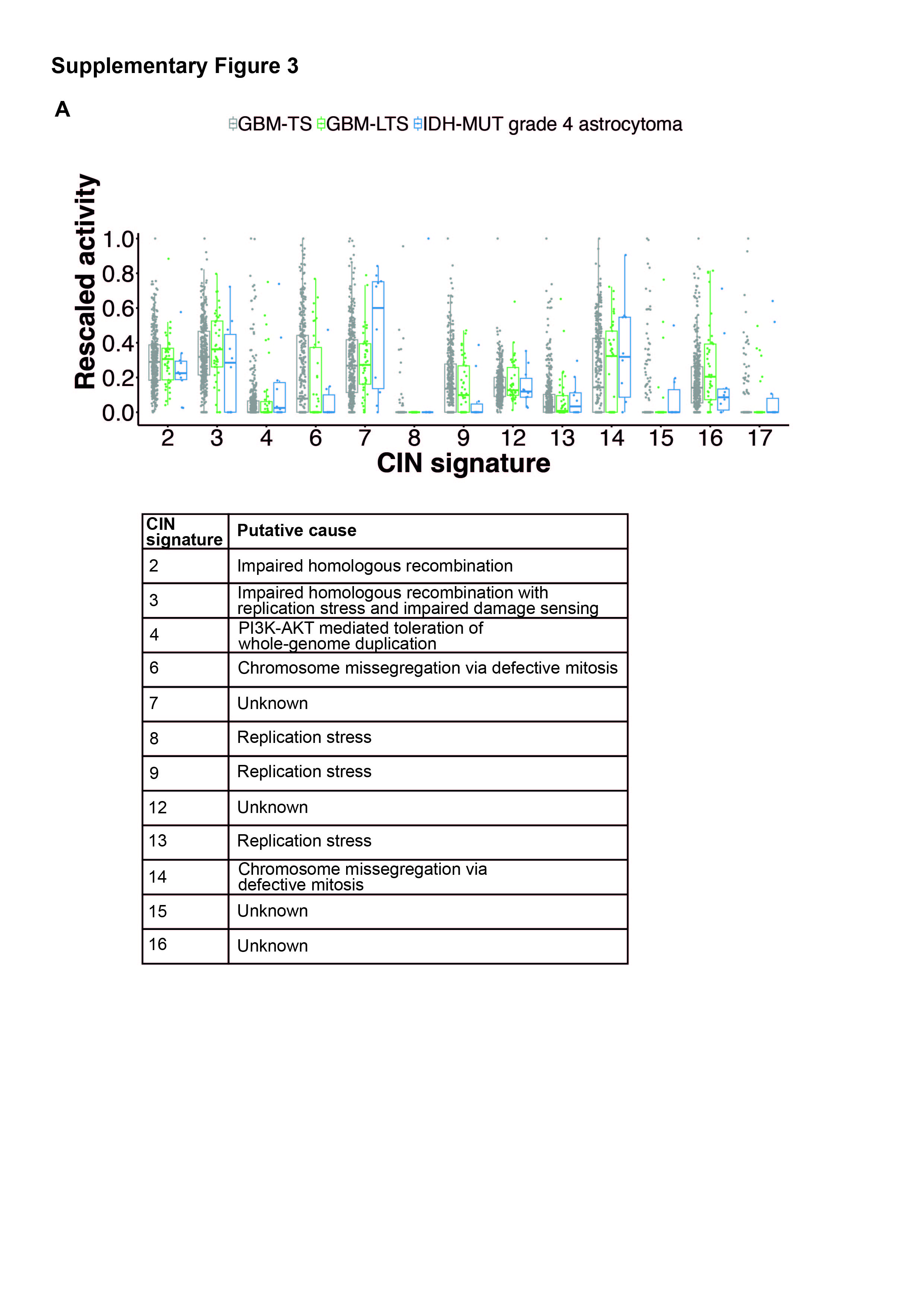

Supplement: Supplementary Figure 3 — (A), Relative activities of 13 CIN signatures that are not significantly different between the 3 groups (GBM-TS, n = 354; GBM-LTS, n = 41; IDH-MUT grade 4 astrocytoma, n = 10). Boxplots summarize rescaled CIN signature activities (Kruskal-Wallis test with Dunn’s post hoc test, corrected for multiple testing by using the Benjamini-Hochberg method, FDR > 0.05). Boxes represent the interquartile range with the median depicted as a bold line and the whiskers extend to 1.5 times the interquartile range from the lower or upper quartile, with datapoints outside this interval being considered as potential outliers. (B), Examples of negative and positive immunohistochemical stains for pRPA-S33, pRPA-S4/S8 and γH2AX. Scale bar, 50 μm. (C-E), Frequency of immunopositive cells. Results represent mean ± SD (one-way ANOVA with Dunnett’s multiple comparisons test). [file Image_3.jpeg]

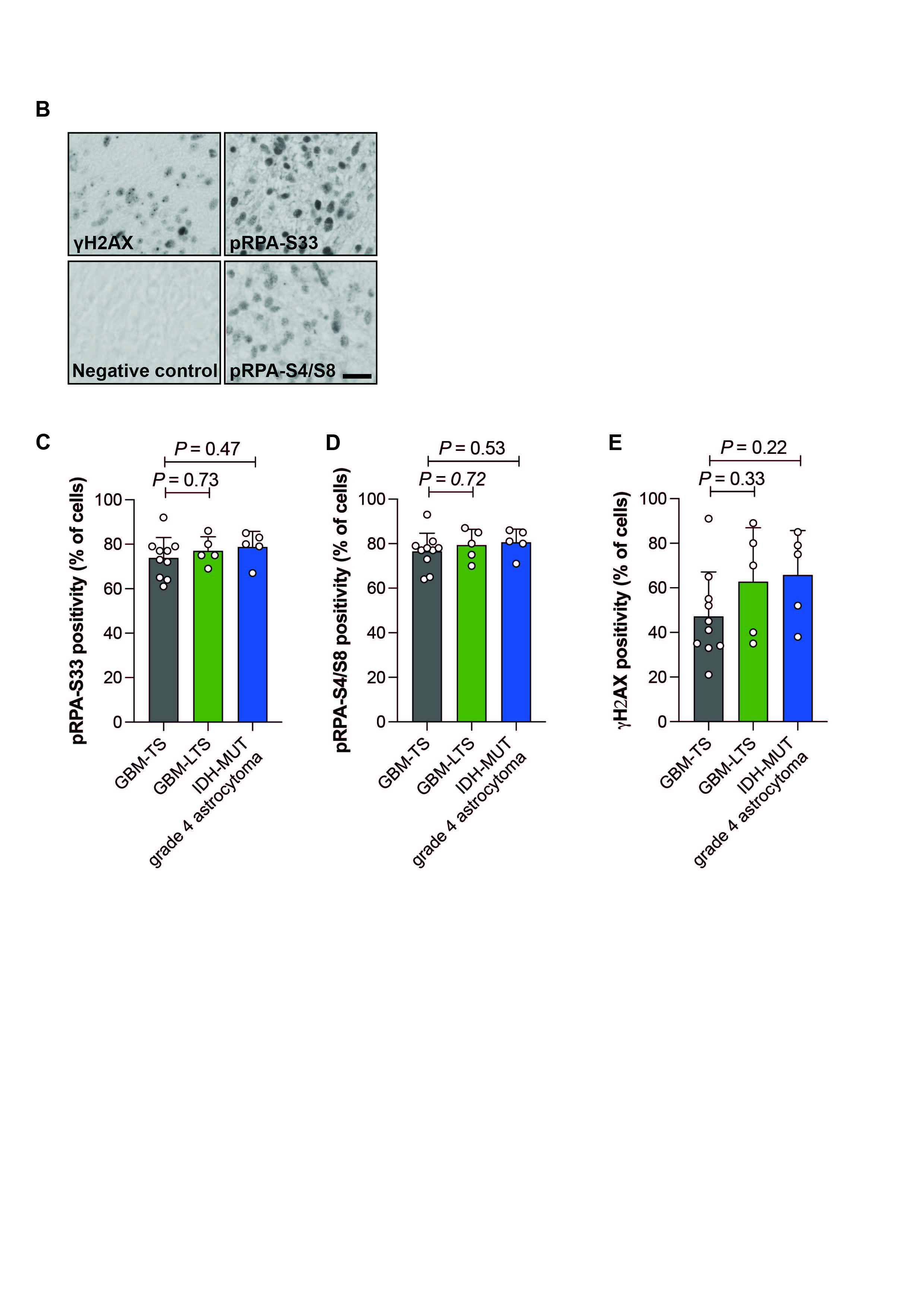

Supplement: Supplementary file 5 [file Image_4.jpeg]
